# Inhibiting the LPS-induced enhancement of mEPSC frequency in superficial dorsal horn neurons may serve as an electrophysiological model for alleviating pain

**DOI:** 10.1038/s41598-019-52405-0

**Published:** 2019-11-05

**Authors:** Chin-Tsang Yang, Shih-Ya Hung, Sheng-Feng Hsu, Iona MacDonald, Jaung-Geng Lin, Sih-Ting Luo, Pei-Lin Lin, Yi-Hung Chen

**Affiliations:** 1 School of Chinese Medicine, China Medical University, Taichung, Taiwan; 2 Department of Leisure Industry and Health Promotion, National Ilan University, Yilan, Taiwan; 3 Department of Medical Research, China Medical University Hospital, Taichung, Taiwan; 4 Graduate Institute of Integrated Medicine, College of Chinese Medicine, China Medical University, Taichung, Taiwan; 5 Department of Chinese Medicine, China Medical University Hospital, Taipei branch, Taipei, Taiwan; 6 Graduate Institute of Acupuncture Science, China Medical University, Taichung, Taiwan; 7 Department of Anesthesiology, National Taiwan University Hospital, Taipei, Taiwan; 8 Chinese Medicine Research Center, China Medical University, Taichung, Taiwan; 9 Department of Photonics and Communication Engineering, Asia University, Taichung, Taiwan

**Keywords:** Pharmacodynamics, Neurophysiology

## Abstract

Pain is a major primary health care problem. Emerging studies show that inhibition of spinal microglial activation reduces pain. However, the precise mechanisms by which microglial activation contributes to nociceptive synaptic transmission remain unclear. In this study, we measured spontaneous synaptic activity of miniature excitatory postsynaptic currents (mEPSCs) in rat spinal cord superficial dorsal horn (SDH, laminae I and II) neurons. Lipopolysaccharide (LPS) and adenosine triphosphate (ATP) increased the frequency, but not amplitude, of mEPSCs in SDH neurons. Microglial inhibitors minocycline and paeonol, as well as an astrocyte inhibitor, a P2Y1 receptor (P2Y1R) antagonist, and a metabotropic glutamate receptor 5 (mGluR5) antagonist, all prevented LPS-induced enhancement of mEPSC frequency. In mouse behavioral testing, minocycline and paeonol effectively reduced acetic acid-induced writhing and LPS-induced hyperalgesia. These results indicate that LPS-activated microglia release ATP, which stimulates astrocyte P2Y1Rs to release glutamate, triggering presynaptic mGluR5 receptors and increasing presynaptic glutamate release, leading to an increase in mEPSC frequency and enhancement of nociceptive transmission in SDH neurons. We propose that these effects can serve as a new electrophysiological model for evaluating pain. Moreover, we predict that pharmacologic agents capable of inhibiting the LPS-induced enhancement of mEPSC frequency in SDH neurons will have analgesic effects.

## Introduction

Pain is a major primary health care problem. In 2011, the National Academy of Medicine reported that chronic pain affects more than 100 million Americans every year – affecting more people than heart disease, cancer and diabetes combined^1^. Associated costs are huge; recent estimates calculate that pain costs the USA half a trillion dollars annually, measured in terms of health care use, loss of wages and impact on quality of life^2^. Issues surrounding current drug treatments for pain include their low analgesic efficacy or dose-limiting adverse effects. Although investigations into pain pathways have clarified that inhibiting microglial activation reduces pain, how exactly this contributes to nociceptive synaptic transmission is not yet fully understood. A better understanding could lead to the design of pharmacotherapies that more effectively target the pain response.

Microglia are generally considered to be the immune cells of the central nervous system (CNS). In the event of acute insults including infection, inflammation, trauma, ischemia, and neurodegeneration, microglia quickly respond and can morphologically transform from a resting state referred to as “ramified” to an active “amoeboid” state^3,4^. Once activated, microglia exhibit a spectrum of phenotypes, releasing both pro- and anti-inflammatory mediators. Proinflammatory mediators include tumor necrosis factor alpha (TNF-α), interleukin 6 (IL-6), and reactive oxidative species^5,6^. Microglia are activated in the spinal cord in almost all animal models of pain, including nerve injury, traumatic injury, inflammatory, and bone cancer pain models^7^. It would be useful to know whether pain-induced microglial activation modulates glutamatergic neurotransmission.

Glutamate is released from sensory afferents in response to acute and more persistent noxious stimuli; fast alpha-amino-3-hydroxy-5-methyl-4-isoxazolepropionic acid (AMPA) glutamate receptor activation is responsible for setting the initial baseline response to noxious stimuli among neurons in the superficial spinal dorsal horn (SDH, laminae I and II)^8^. Peripheral tissue injury during the critical period of early life can facilitate nociceptive transmission by enhancing glutamate release onto the SDH neurons^8,9^. Presynaptic glutamate release can be measured by the frequency of miniature excitatory postsynaptic currents (mEPSCs) and postsynaptic responsiveness by mEPSC amplitude^10^. Surgical incision through the skin and muscle of the hindlimb in Sprague-Dawley rat pups (postnatal day 3 [P3] or P10) selectively increases the frequency, but not amplitude, of glutamatergic mEPSCs recorded 2–3 days after injury in the SDH^11^. Following spared nerve injury, the frequency of mEPSCs doubled, indicating heightened glutamate release from primary afferents or spinal interneurons in lamina II of the dorsal horn.

In hippocampal slices, lipopolysaccharide (LPS)-induced activation of microglial cells stimulates astrocytes, which in turn increase glutamatergic transmission and mEPSC frequency^12^. This mechanism may have important physiopathological relevance in responses to pain. In SDH neurons of the spinal cord, the elevation of CC chemokine ligand 2 (CCL2) levels induced by peripheral nerve injury causes thermal hyperalgesia and facilitates spinal nociceptive (glutamatergic) transmission. Minocycline, an inhibitor of microglial activation, inhibits CCL2-induced heat hyperalgesia and blocks the enhancement of glutamatergic transmission and mEPSC frequency^13^.

While some studies have reported a reduction in pain with the inhibition of spinal microglial activation^14–17^, the precise mechanisms by which microglial activation contributes to nociceptive synaptic transmission remain unclear. Our novel electrophysiological pain model hypothesized that therapeutic agents capable of inhibiting LPS-induced enhancement of mEPSC frequency in SDH neurons exert an analgesic effect by blocking microglial activation and nociceptive transmission. In order to confirm this hypothesis, we investigated pathological mechanisms and selected the microglial inhibitors, minocycline and paeonol^18,19^, to test whether these agents inhibit the LPS-induced enhancement of mEPSCs. We also tested whether minocycline and paeonol reduce acetic acid-induced acute pain levels and LPS-induced hyperalgesia.

## Results

### LPS increased the frequency, but not the amplitude, of AMPA glutamate receptor-mediated mEPSCs in superficial spinal dorsal horn neuron*s*

**Figure 1:**
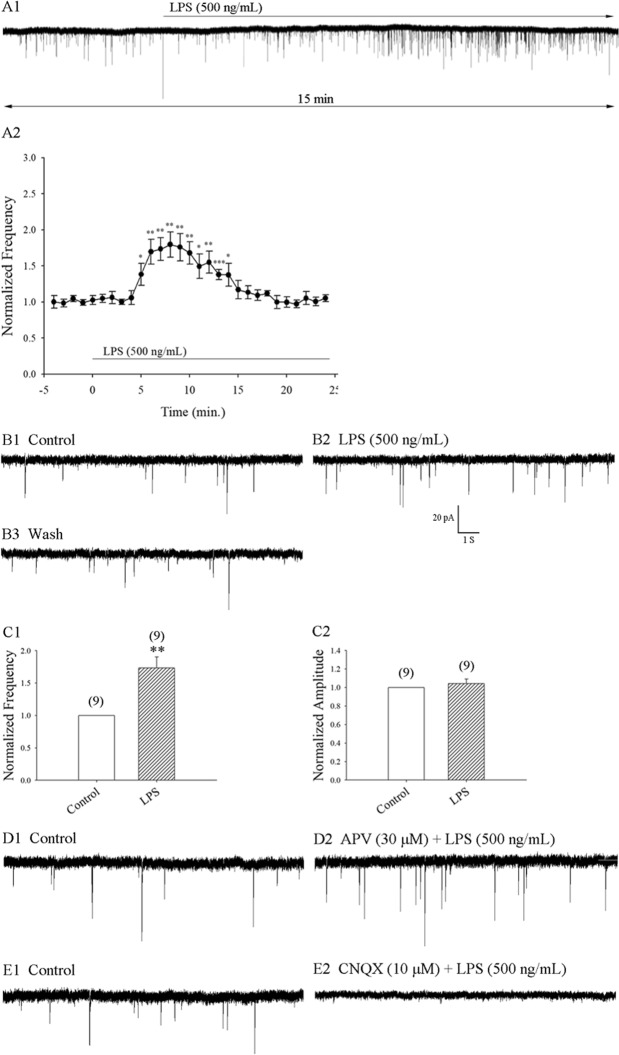
LPS increased the frequency, but not amplitude, of mEPSCs in superficial SDH neurons. (**A1**) A continuous 15-min trace recording showing the mEPSCs before and LPS perfusion in an SDH neuron recorded in the presence of bicuculline 10 µM and TTX 1 µM. (**A2**) Timecourse of average mEPSC frequency change in response to LPS (9 neurons from 9 rats). (**B1**) Control: sample traces of mEPSCs recorded in another neuron under the same condition of (**A1**). (**B2**) mEPSCs from (**B1**) recorded at 8 min of exposure to LPS 500 ng/mL. B3: 20 min after washing from (**B2**). (**C1,C2**) Average frequency and amplitude values of mEPSCs over a 5-min period at 6–10 min of exposure to LPS 500 ng/mL (9 neurons from 9 rats) compared with control levels. (**D1,E1**) Control: sample traces of mEPSCs. (**D2**) mEPSCs from (**D1**) recorded at 8 min of exposure to LPS with APV. The neurons were pretreated with APV 30 μM 15 min before LPS perfusion. (**E2**) mEPSCs from (**E2**) recorded after 8 min of exposure to LPS with CNQX. Neurons were pretreated with CNQX 10 μM 5 min before LPS perfusion.

Spinal cord microglia are activated in almost all animal models of pain. Whether microglial activation increases glutamatergic neurotransmission in dorsal horn neurons is unclear. In this study, we examined whether LPS enhances glutamatergic neurotransmission by measuring the frequency and amplitude of mEPSCs in rat SDH neurons. All experiments were recorded in voltage-clamp mode. mEPSCs were isolated in the presence of the Na^+^ channel blocker tetrodotoxin (TTX, 1 µM) and the GABA receptor antagonist bicuculline (10 μM) and maintained at a holding potential of −70 mV at room temperature. In control neurons, the mean resting membrane potential (RMP), series resistance (Rs) and membrane resistance values were −61.4 ± 3 mV, 11.2 ± 0.9 MΩ, and 576.7 ± 43.5 MΩ, respectively; the mEPSC frequency and amplitude were 0.65 ± 0.15 Hz and 20.1 ± 2.3 pA, respectively (9 neurons from 9 rats). LPS increased the frequency of mEPSCs in superficial SDH neurons (Fig. 1A1). This increase was transient, starting at 5 min and lasting around 10 min before returning to control levels, as shown in Fig. 1A2. Average mEPSC frequency was increased from baseline to 173.4% ± 17.0% over a 5-min period, starting after 6 min of LPS perfusion 500 ng/mL (9 neurons from 9 rats), as shown in Fig. 1B2,C1. After a washout period of 15–20 min, 5 of these 9 neurons were tested in a second LPS challenge, which demonstrated a reproducible increase in mEPSC frequency. The average mEPSC frequency over a 5-min period at 6–10 min of LPS exposure was significantly increased to 166.1% ± 13.6% (5 neurons from 5 rats; p = 0.008) of control levels. In contrast, LPS perfusion had no effect on the amplitude of mEPSCs (n = 9; p = 0.39; see Fig. 1B2,C2). Use of the LPS concentration of 500 ng/mL is based on previous studies that recorded LPS-mediated increases in EPSC frequency in the brain^12,20^.

When *N*-methyl-D-aspartate (NMDA) transmission was blocked by the NMDA glutamate receptor antagonist APV (30 µM), the LPS-mediated increase in mEPSC frequency was unchanged (Fig. 1D2). When perfused with the AMPA/kainite glutamate receptor antagonist, CNQX (10 µM), the mEPSCs were abolished (Fig. 1E2). It appears that LPS-mediated increases in mEPSC frequency involve AMPAergic transmission.

The frequency and amplitude of mEPSCs varied substantially between neurons. We therefore calculated the normalized frequency and amplitude for each neuron.

### LPS-mediated increases in mEPSC frequency occurs through the activation of microglia

**Figure 2:**
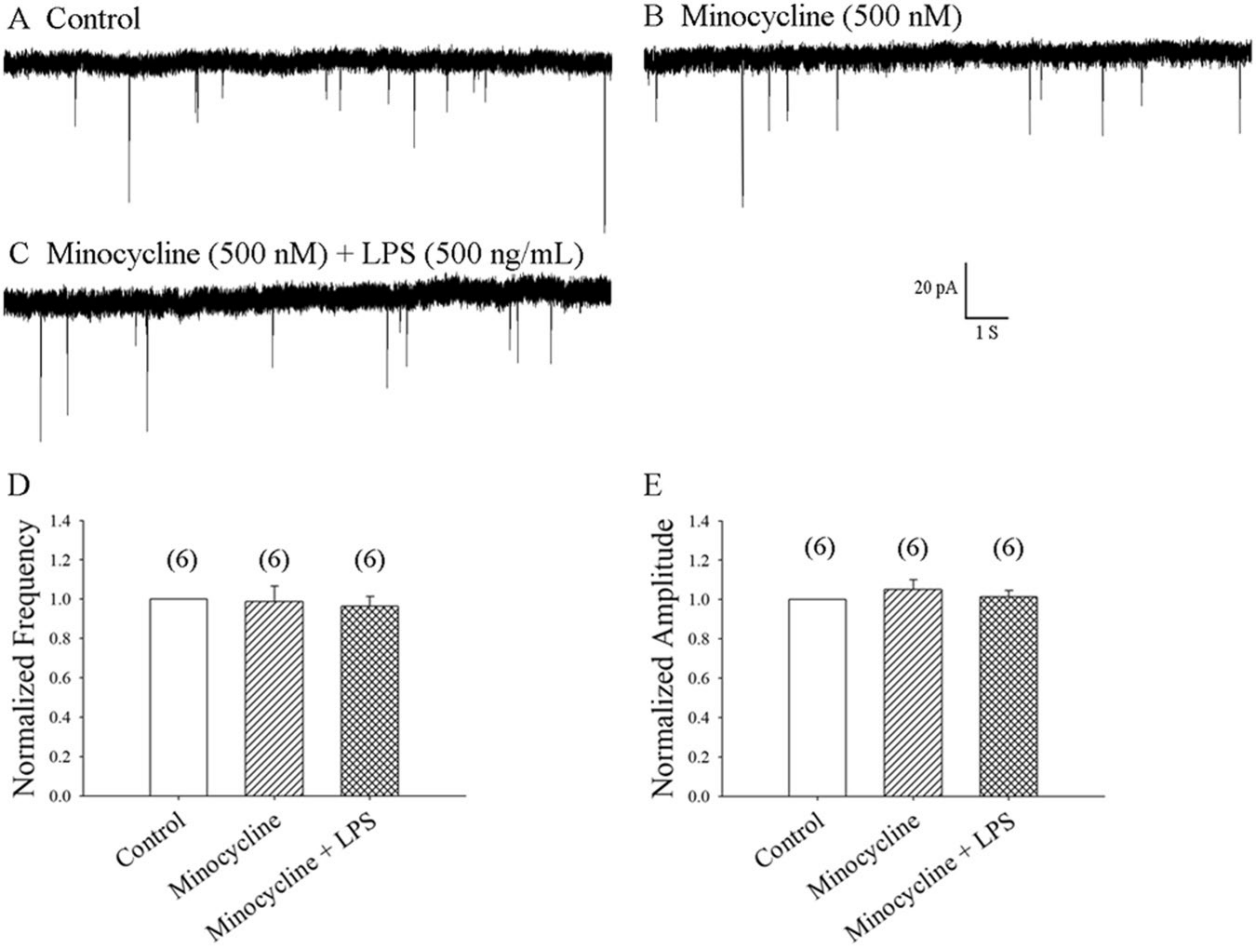
Pretreatment with minocycline prevented LPS-induced enhancement of mEPSC frequency. (**A)** Control: sample traces of mEPSC recordings from a neuron in the presence of bicuculline 10 µM and TTX 1 µM. (**B**) mEPSCs from A recorded after 20 min of exposure to minocycline 500 nM. (**C**) mEPSCs from B recorded at 8 min of exposure to LPS 500 ng/mL. (**D**,**E**) Average frequency and amplitude values of mEPSCs over a 5-min period before and at 6–10 min of exposure to minocycline with or without LPS, respectively (6 neurons from 5 rats).

Minocycline is commonly used to inhibit microglial activation. We examined the role of microglia in LPS-mediated increases in mEPSC frequency. Following previously established methods^21^, we perfused spinal slices with minocycline 500 nM for 20 min. Although basal mEPSC frequency and amplitude was not significantly changed by minocycline, LPS-induced increases in mEPSC frequency were prevented, as shown in Fig. 2 (6 neurons from 5 rats). It appears that LPS-mediated increases in mEPSC frequency occur through the activation of microglia.

### Microglia activation triggers astrocyte-mediated modulation of mEPSCs

**Figure 3:**
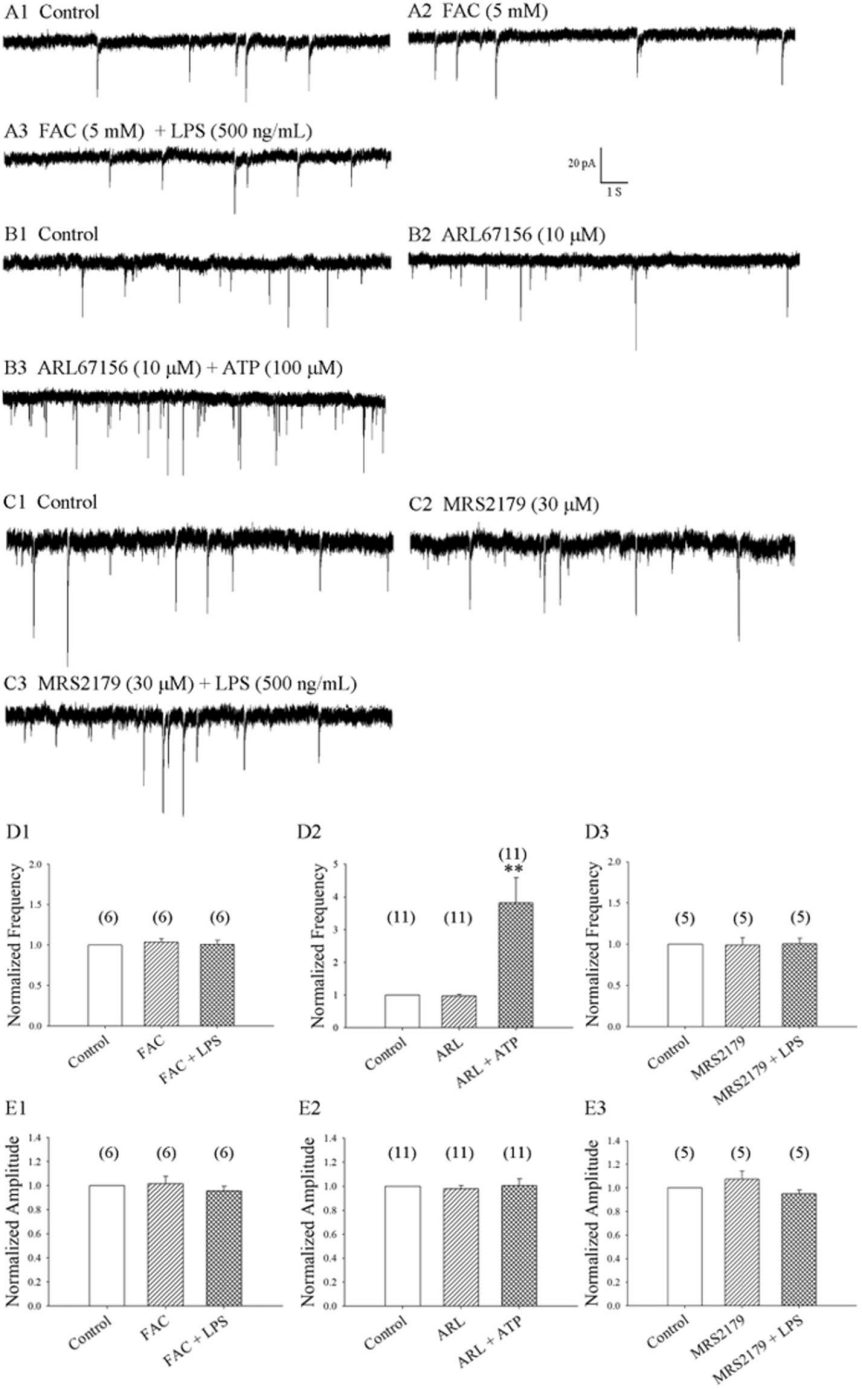
Pretreatment with the astrocyte inhibitor, FAC, and the P2Y1 antagonist, MRS2179, prevented LPS-induced enhancement of mEPSC frequency, while ATP increased the frequency of mEPSCs. (**A1**,**B1**,**C1**) Control: sample traces of mEPSCs recorded in a neuron in the presence of bicuculline 10 µM and TTX 1 µM. (**A2**,**B2**,**C2**) mEPSCs from (**A1**,**B1**,**C1**), recorded after 20 min of exposure to FAC (5 mM), ARL67156 (10 μM) and MRS2179 (30 μM), respectively. (**A3**,**C3**) mEPSCs from (**A2**,**C2**) recorded at 8 min of exposure to LPS 500 ng/mL. (**B3**) mEPSCs from B2 recorded at 8 min of exposure to ATP 100 μM. (**D1**,**D2**,**D3**,**E1**,**E2**,**E3**) Average frequency (**D1**,**D2**,**D3**) and amplitude (**E1**,**E2**,**E3**) values of mEPSCs recorded over a 5-min period before and at 6–10 min of exposure to FAC with or without LPS (6 neurons from 6 rats), to ARL67156 with or without ATP (11 neurons from 11 rats), and to MRS2179 with or without LPS (5 neurons from 5 rats), respectively.

It is widely accepted that astrocytes release gliotransmitters^22^, which modulate neuronal excitability and neurotransmission^23^. We investigated whether astrocytes are involved in the LPS-mediated increase in mEPSC frequency. We perfused slices for 20 min with fluoroacetate (FAC), a known blocker of astrocytic function, at a concentration of 5 mM. These conditions are moderate compared with those in other studies^24–26^. We found that FAC 5 mM did not significantly change the basal mEPSC frequency and amplitude, but the LPS-induced increase in mEPSC frequency was prevented, as shown in Fig. 3A,D1,E1 (6 neurons from 5 rats). It appears that LPS-mediated modulation of mEPSCs requires astrocyte involvement.

In mixed cultured microglia and astrocytes, it has been established that LPS-activated microglia rapidly release small amounts of ATP that recruit astrocytes that amplify ATP release^12^. ATP reportedly increases sEPSC frequency in lamina V neurons^27^. We examined whether ATP is involved in LPS-induced enhancement of mEPSC frequency in SDH neurons. We found that a bath application of ATP 100 μM significantly increased mEPSC frequency by nearly three-fold (381.9 ± 76.7%; p = 0.004; 11 neurons from 8 rats) (Fig. 3B,D2,E2) that lasted for 5 min, whereas mEPSC amplitude was unaffected in rat SDH neurons. These results are supported by a previous report, in which bath application of ATP 100 μM increased the mEPSC frequency in lamina I neurons^28^. This suggests that LPS-activated microglia and astrocytes release ATP, which may modulate the enhancement of mEPSC frequency. In our experiment, 100 μM ATP was applied in the presence of 10 μM ARL67156, an ecto-ATPase inhibitor that prevents ATP metabolism^27,28^.

Extracellular ATP is able to signal through different purinergic receptors, which include ionotrophic P2Xs and G protein-coupled P2Ys^29^. P2Y1R activation is responsible for the ATP-induced glutamate efflux from astrocytes^30^. Astrocytes release glutamate^22^, which modulates neuronal excitability and neurotransmission^23^. We found that basal mEPSC frequency and amplitude were not significantly altered by the P2Y1R antagonist, MRS2179 (30 μM), whereas MRS2179 did prevent LPS-induced increases in mEPSC frequency, as shown in Fig. 3C,D3,E3 (5 neurons from 5 rats). It appears that LPS modulates mEPSCs via the P2Y1R regulatory pathway.

### Glutamate released by astrocytes facilitates activation of presynaptic mGluR5 activation and increases neuronal glutamate transmission

**Figure 4:**
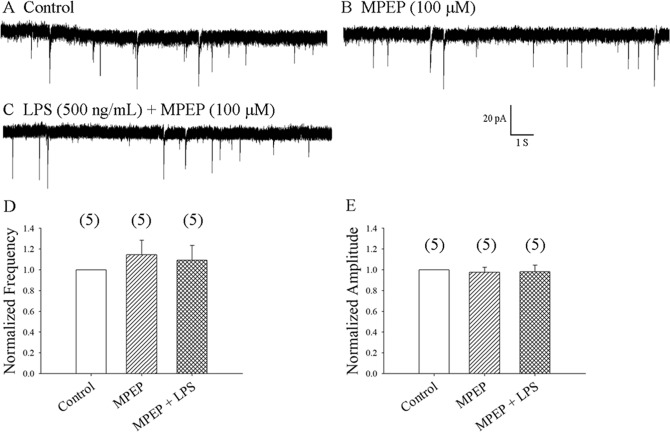
Pretreatment with the mGluR5 antagonist, MPEP, prevented the LPS-induced enhancement of mEPSC frequency. (**A)** Control: sample traces of mEPSCs recorded in a neuron in the presence of bicuculline 10 µM and TTX 1 µM. (**B**) mEPSCs from A recorded after 20 min of exposure to MPEP 100 μM. (**C**) mEPSCs from (B) recorded at 8 min of exposure to LPS 500 ng/mL. (**D**,**E**) Average frequency and amplitude values of mEPSCs over a 5-min period before and at 6–10 min of exposure to MPEP with or without LPS, respectively (5 neurons from 5 rats).

Glutamate released by astrocytes can facilitate neurotransmitter release by activating presynaptic group I metabotropic glutamate receptors (mGluRs)^22,31–33^. In hippocampus neurons, the mGluR5 antagonist 2-methyl-6-(phenylethynyl) pyridine (MPEP) (100 μM) abolished LPS-induced increases in mEPSC frequency^12^. We found that MPEP at 100 μM did not significantly change basal mEPSC frequency and amplitude in SDH neurons, but MPEP did abolish the effect of LPS application, as shown in Fig. 4 (5 neurons from 5 rats). This indicates that LPS modulates mEPSCs via the presynaptic mGluR5 pathway.

### Pretreatment with the microglial inhibitor, paeonol, prevented the LPS-induced enhancement of mEPSC frequency

**Figure 5:**
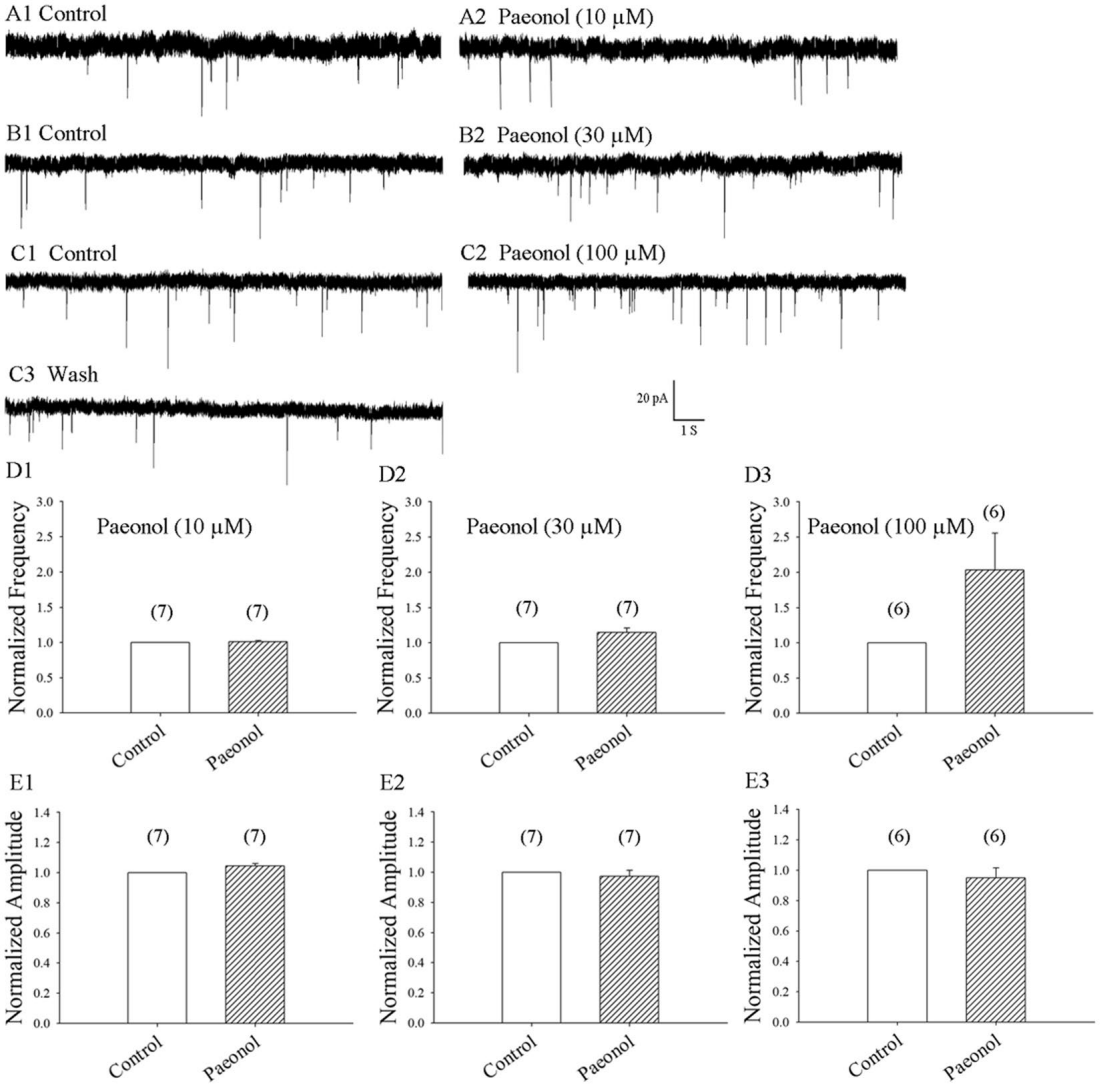
Paeonol (10 µM) did not change the basal EPSC frequency and amplitude of mEPSCs. (**A1**,**B1**,**C1**) Control: sample traces of mEPSCs recorded in a neuron in the presence of bicuculline 10 µM and TTX 1 µM. (**A2**,**B2**,**C2**) mEPSCs recorded after 10 min of exposure to paeonol 10 µM, paeonol 30 µM and paeonol 100 µM from (**A1**,**B1**,**C1**), respectively. (**C3**) 10 min after washing from (**C2)**. (**D1**,**D2**,**D3**,**E1**,**E2**,**E3**) Average mEPSC frequency and amplitude values over a 5-min period before and at 10 min of exposure to paeonol 10 µM, 30 µM, or 100 µM, respectively.

**Figure 6:**
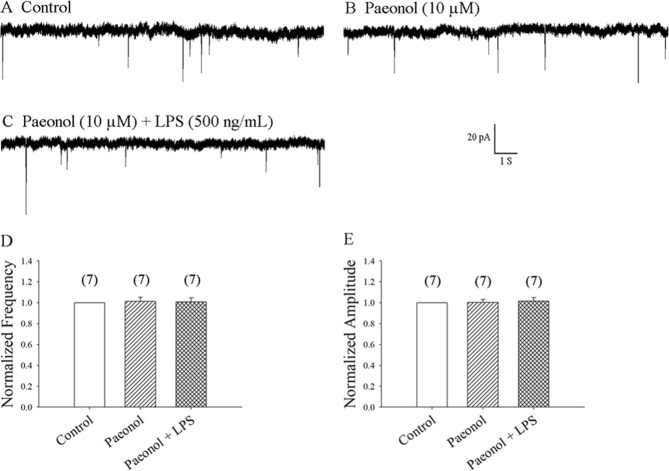
Pretreatment with paeonol prevented the LPS-induced enhancement of mEPSC frequency in superficial SDH neurons. (**A**) Control: mEPSCs recorded in the presence of bicuculline 10 µM and TTX 0.5 µM in a neuron. (**B**) mEPSCs recorded from A after 15 min of exposure to paeonol 10 µM. (**C**) mEPSCs recorded from (B) at 8 min of LPS 500 ng/mL. (**D**,**E**) Average mEPSC frequency and amplitude values over a 5-min period before and at 6–10 min of exposure to paeonol with or without LPS, respectively (7 neurons from 7 rats).

Paeonol might act as a microglial inhibitor^18,19^. We examined whether paeonol prevents LPS-induced enhancement of mEPSC frequency. Our previous report found that paeonol (>30 µM) increased the frequency of mEPSCs, but had no effect on amplitude in rat hippocampal CA1 neurons^34^. We therefore investigated whether different concentrations of paeonol modulate the frequency or amplitude of mEPSCs in rat SDH neurons. Perfusion of SDH neurons with paeonol at the concentrations of 10, 30 and 100 µM resulted in mEPSC frequencies of 102.9% ± 1.9% (7 neurons from 7 rats; p = 0.60) (Fig. 5A2,D1), 109.9% ± 6.6% (7 neurons from 7 rats; p = 0.07) (Fig. 5B2,D2) and 197.8% ± 52.3% (6 neurons from 6 rats; p = 0.10) (Fig. 5C2,D3), respectively, compared with values from control neurons. Similarly, perfusion with paeonol at the concentrations of 10, 30 and 100 µM did not significantly affect mEPSC amplitudes, which were 102.0% ± 3.7% (7 neurons from 7 rats; p = 0.50), 98.9% ± 2.9% (7 neurons from 7 rats; p = 0.60) and 96.2% ± 4.2% (6 neurons from 6 rats; p = 0.60), respectively, compared with controls (Fig. 5E1–E3). These results suggest that paeonol at the concentrations of 10 µM clearly had no effects on the basal frequencies or amplitudes of mEPSCs in rat SDH neurons. We then investigated whether paeonol at 10 µM modulates LPS-induced increases in mEPSC frequency. We found that pretreatment with paeonol 10 µM prevents the LPS-induced increase in mEPSC frequency (7 neurons from 7 rats), as shown in Fig. 6.

### Minocycline and paeonol reduce acute acetic acid-induced visceral pain

**Figure 7:**
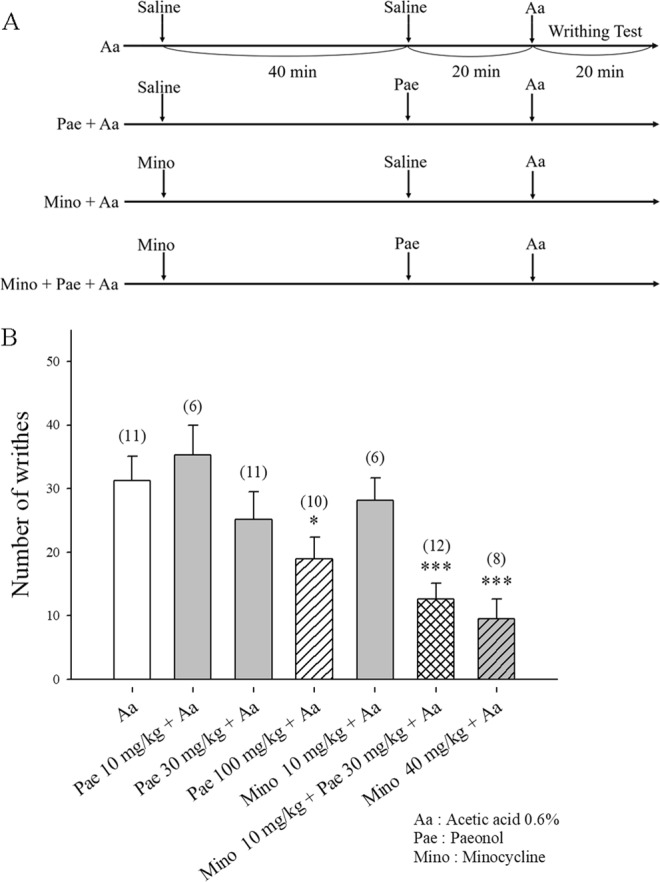
Administration of minocycline or paeonol inhibits acetic acid-induced abdominal pain. (**A**) Timeline depicting administration schedule for minocycline and paeonol. The number of writhes was counted for a continuous 15-min period, starting at 5 min after i.p. administration of acetic acid 0.6%. Mice were pretreated with i.p. minocycline 10 or 40 mg/kg 1 h prior to acetic acid 0.6%, or with i.p. paeonol (10, 30 or 100 mg/kg) 20 min before acetic acid 0.6%. B: Acetic-induced writhing was significantly reduced by paeonol (100 mg/kg), minocycline (10 mg/kg) + paeonol (30 mg/kg), and by minocycline (40 mg/kg). *p < 0.05, ***p < 0.001; for comparisons with controls (acetic acid only).

We counted the number of abdominal constrictions or writhes for a continuous 15-min period, starting at 5 min after mice were administered acetic acid 0.6% (Fig. 7). The number of acetic acid-induced writhes was 31.3 ± 3.8 in the control group. Paeonol 10 mg/kg had no effect upon acetic acid-induced abdominal contractions (n = 6; p = 0.5). Paeonol 30 mg/kg or minocycline 10 mg/kg slightly inhibited acetic acid-induced abdominal contractions; writhing decreased to 76.4% (n = 11; p = 0.3) and 85.5% (n = 6; p = 0.6) of controls, respectively. Pretreatment with minocycline 10 mg/kg plus paeonol 30 mg/kg significantly decreased writhing to 38.2% of controls (n = 12; p = 0.0005). Pretreatment with paeonol 100 mg/kg or minocycline 40 mg/kg also significantly decreased writhing to 57.7% (n = 10; p = 0.03) and 28.8% (n = 8; p = 0.0002) of controls, respectively.

### Minocycline and paeonol block LPS-induced hyperalgesia

**Figure 8:**
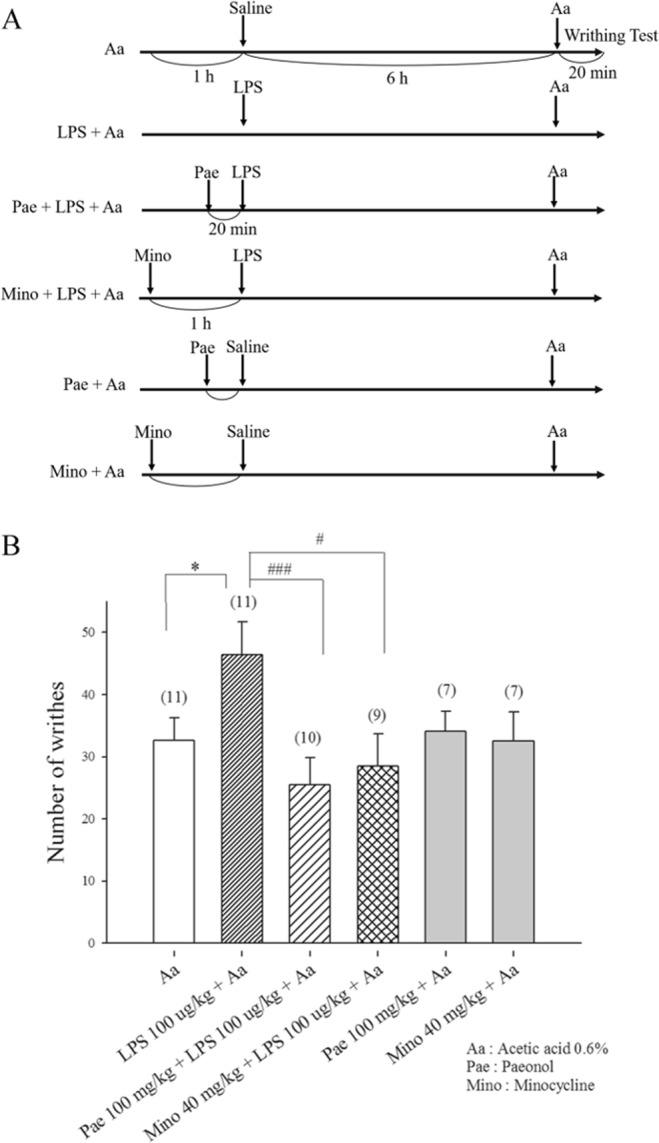
Administration of minocycline or paeonol reduced LPS-induced hyperalgesia. (**A**) Timeline depicting administration schedule for minocycline, paeonol and LPS. Mice received i.p. minocycline (40 mg/kg) 1 h prior or i.p. paeonol (100 mg/kg) 20 min prior to LPS (100 µg/kg) administration. At 6 h after LPS administration, the mice were subjected to acetic acid-induced abdominal constriction (writhing) testing, to determine hyperalgesic responses to LPS in the presence or absence of paeonol or minocycline. (**B**) When pretreated with minocycline at 1 h or paeonol at 20 min before LPS administration, the number of writhes induced by acetic acid 0.6% was decreased to 61.5% and 54.8%, respectively, compared to the LPS plus acetic acid group. Pretreatment with paeonol or minocycline 6 h prior to acetic acid injection did not reduce hyperalgesia. *p < 0.05; for comparisons with the acetic acid-only group, ^#^p < 0.05; ^###^p < 0.001; for comparisons with the acetic acid plus LPS group.

Previous reports indicated that intraperitoneally-injected LPS evoked time-dependent hyperalgesia, with a maximal change in behavioral responses occurring at 6 h after LPS treatment^35,36^. We examined whether LPS treatment for 6 h enhances acetic acid-induced visceral pain, and whether minocycline and paeonol reduce LPS-induced hyperalgesia. In the control group of this study, the number of writhes induced by acetic acid 0.6% was 32.6 ± 3.6 (n = 11). When mice were pretreated with LPS (100 µg/kg) 6 h before acetic acid injection, the number of writhes induced by acetic acid 0.6% was 46.5 ± 5.2, which was significantly increased to 142.6% (n = 11; p = 0.04), compared to controls (see Fig. 8). This result indicates that LPS induces hyperalgesia. Pretreatment with paeonol (100 mg/kg) at 20 min or minocycline (40 mg/kg) at 1 h before LPS (100 µg/kg) administration significantly reduced writhing induced by acetic acid 0.6%; after 6 h, the number of writhes was decreased to 54.8% (n = 10; p = 0.006) and 61.5% (n = 9; p = 0.03), respectively, compared to the LPS plus acetic acid group. This result indicates that minocycline and paeonol reduce LPS-induced hyperalgesia. While pretreatment with minocycline (40 mg/kg) or paeonol (100 mg/kg) 6 h prior to acetic acid injection did not change acetic acid-induced visceral pain. The numbers of writhes were 99.8% (n = 8; p = 0.8) and 104.6% (n = 8; p = 0.9) respectively, compared to controls (see Fig. 8). After 6 h, minocycline (40 mg/kg) or paeonol (100 mg/kg) had lost their analgesic efficacy.

### Injection of LPS activated microglia and astrocytes in spinal cord

The T5–L2 spinal cord segments are involved in pain relating to intraperitoneal acetic acid injection^37^. In the present study, we performed immunohistochemistry evaluations of astrocytes and microglia from L1–L2 segments. Astrocytes were stained with glial fibrillary acidic protein (GFAP). After injection of LPS (100 µg/kg), minimal changes in cell morphology were observed at 2 h (Supplementary Fig. 1, middle photographs), whereas prominent morphological changes were observed at 6 h, with a higher density of astrocytes, more dendrites and larger cell bodies (Supplementary Fig. 1, right-hand column). Microglia were stained with ionized calcium binding adaptor molecule 1 (Iba1). Two h after injection of LPS, very minimal morphological changes were observed, with the microglia resembling those of the control condition (Supplementary Fig. 2, middle column). However, by 6 h, cells appeared to have larger cell bodies with more dendrites (Supplementary Fig. 2, right-hand column). After LPS (100 µg/kg) injection, Western blot analysis of mouse spinal cord tissue revealed mean relative Iba1 expression levels of 97.9% at 2 h (n = 6, p = 0.63) and 111.8% at 6 h (n = 6, p = 0.01) (Supplementary Fig. 3A), compared to controls; corresponding mean relative GFAP expression levels were 103.7% (n = 9, p = 0.85) and 150% (n = 9, p = 0.04), (Supplementary Fig. 3B), respectively, compared to controls. Microglia and astrocytes were activated at 6 h after LPS treatment in mice spinal cord.

## Discussion

Most pain information carried by the primary afferent fibers A${\rm{\delta }}$ and C is processed and integrated by SDH neurons in laminae I–II of the spinal cord; a smaller portion of information reaches deeper (lamina V) dorsal horn neurons^8,13^. Acute and more persistent noxious stimuli, signaled by the release of glutamate from the central terminals of nociceptors, generate EPSCs in second-order SDH neurons. This occurs primarily through the activation of postsynaptic AMPA glutamate receptors^38^.

Microglial activation is commonly found in the spinal cord in almost all animal models of pain. Whether pain-mediated microglial activation increases glutamatergic neurotransmission in the dorsal horn neurons is not fully understood. In the mouse hippocampus, LPS-induced microglia activation enhances glutamatergic neurotransmission^12^. It is generally believed that mEPSCs are mediated by glutamate AMPA/kainite receptors and that changes in the frequency and amplitude of mEPSCs are mediated by pre- and postsynaptic mechanisms, respectively^39^. In this study, we examined whether LPS enhances glutamatergic neurotransmission by measuring the frequency and amplitude of mEPSCs in rat SDH neurons. Perfusion with LPS increased the frequency of mEPSCs, but not the amplitude (Fig. 1B2,C1,C2). APV (30 µM), an NMDA glutamate receptor antagonist, could not prevent LPS-mediated increases in mEPSC frequency (Fig. 1D2). In contrast, perfusion with CNQX (10 µM), an AMPA glutamate receptor antagonist, abolished the LPS-mediated increases in mEPSCs (Fig. 1E2). These results are consistent with earlier research in hippocampal neurons of mice^12^ and suggest that the microglial activation induced by LPS increases presynaptic glutamatergic neurotransmission and involves AMPAergic transmission.

While several studies have reported reductions in pain via the inhibition of spinal microglial activation, the precise synaptic mechanisms have not been fully understood. We investigated whether blocking microglial activation inhibits LPS-induced increases in the frequency of mEPSCs. We found that pretreatment with minocycline 500 nM 20 min prior to LPS administration did not significantly change basal mEPSC frequency or amplitude, but it did prevent LPS-induced increases in mEPSC frequency, as shown in Fig. 2. These results support the hypothesis that LPS-mediated increases in mEPSC frequency occur through the activation of microglia. Modulating glutamate release or uptake after peripheral nerve injury should be an important target in the management of hyperalgesia and chronic neuropathic pain^13,36^. It is known that blocking microglial activation with minocycline inhibits CCL2-induced heat hyperalgesia and blocks CCL2-induced enhancement of nociceptive (glutamatergic) transmission, as well as any increase in frequency of mEPSCs^13^. We hypothesized that inhibiting spinal microglial activation plays a critical role in the reduction of pain, by blocking the enhancement of nociceptive transmission and mEPSC frequency.

Recent electrophysiology and optical imaging analyses have provided strong evidence that astrocytes respond to neurotransmitters and release chemical transmitters called “gliotransmitters”^40,41^, including glutamate, D-serine, or ATP, which modulate neuronal activity, synaptic transmission and plasticity^22,31,42^. We therefore investigated whether astrocytes are involved in the LPS-enhanced frequency of mEPSCs. We found that 20 min of perfusion with FAC (5 mM), a recognized blocker of astrocytic function, did not significantly change basal mEPSC frequency or amplitude, but it did prevent LPS-induced increases in mEPSC frequency, as shown in Fig. 3A,D1,E1. Astrocytes seem to be involved in LPS-induced increases of mEPSC frequency.

Activated microglia release numerous mediators, amongst which ATP and TNF-α reportedly increase EPSC frequency in spinal cord neurons^27,28,43,44^. This modulation is independent of TNF-α, because LPS-induced enhancement of EPSC frequency was not significantly different from TNF-α KO mice compared with WT mice in hippocampal neurons^12^. Therefore, LPS-induced enhancement of mEPSC frequency may be mediated by the binding of ATP released from microglia.

Peripheral nerve pain leads to microglial and astrocytic activation in the dorsal horn^45,46^. In mixed cultured microglia and astrocytes, it has been established that LPS-activated microglia rapidly release small amounts of ATP that recruit astrocytes which amplify ATP release through the astrocyte P2Y regulatory pathway^12^. Extracellular ATP is able to signal through different purinergic receptors, which include ionotrophic P2Xs and G protein-coupled P2Ys^29^. P2Y1R activation is responsible for the ATP-induced glutamate efflux from astrocytes^30^. Astrocytes release glutamate^22^, which modulates neuronal excitability and neurotransmission^23^.

In SDH neurons, we found that ATP 100 μM increased the frequency, but not the amplitude, of mEPSCs, as shown in Fig. 3B,D2,E2. We also found that the P2Y1R antagonist MRS2179 (30 μM) did not significantly change basal mEPSC frequency or amplitude, but it did prevent LPS-induced increases in mEPSC frequency, as shown in Fig. 3C,D3,E3. LPS-induced modulation of mEPSCs appears to occur via the ATP and P2Y1 regulatory pathways.

P2Y1Rs are only expressed by astrocytes and interneurons^47–49^. We found that inhibiting astrocytic function can fully abolish LPS-induced increases in mEPSC frequency. We therefore focused on P2Y1Rs in astrocytes. P2Y1R activation is responsible for the ATP-induced glutamate efflux from astrocytes^30^. Astrocyte-mediated release of glutamate facilitates neurotransmitter release by activating presynaptic group I mGluRs^22,31–33^. The mGluR5 antagonist, MPEP (100 μM), has been shown to abolish LPS-induced increases in mEPSC frequency in hippocampal neurons^12^. We therefore examined the effects of MPEP in SDH neurons. We found that the mGluR5 antagonist MPEP (100 μM) did not significantly change basal mEPSC frequency or amplitude, but it did abolish the effect of LPS application in SDH neurons, as shown in Fig. 4. This indicates that LPS modulates mEPSCs via the presynaptic mGluR5 pathway in SDH neurons.

When we combine the results from Fig. 1 through Fig. 4, we suggest that LPS activation prompts spinal microglia to rapidly release ATP and stimulate astrocyte P2Y1Rs to release glutamate, which then triggers presynaptic mGluR5 receptors and increases presynaptic glutamate release. This mechanism is the same as that previously observed in hippocampal neurons by Pascual and colleagues^12^. However, we performed further experiments with ATP and confirmed the role of ATP in this pathway. We also demonstrated that this mechanism occurs in the spinal cord and may have important physiopathological relevance during pain disorders, considering the fact that microglial activation and enhanced nociceptive glutamatergic transmission occur early in most pain models.

We hypothesized that drugs capable of inhibiting the LPS-induced enhancement of mEPSC frequency in SDH neurons would have an analgesic effect, by blocking microglial activation and subsequently inhibiting nociceptive transmission via the inhibition of glutamate release. We selected minocycline and paeonol as test drugs to determine whether microglial inhibitors inhibit the LPS-induced enhancement of mEPSC frequency. We also tested whether minocycline and paeonol reduce acid-induced acute visceral pain and LPS-induced hyperalgesia.

We have shown that minocycline prevents LPS-induced increases in mEPSC frequency (see Fig. 2). We also found that pretreatment with paeonol, a potential microglial inhibitor^18,19^, prevents LPS-induced increases in mEPSC frequency, as shown in Fig. 6. These results suggest that blocking microglial activation prevents the enhancement of mEPSC frequency.

Intraperitoneal injection of diluted solutions of acetic acid is a well-established rodent model for tonic visceral pain, used to determine the analgesic activity of drugs^50^. The writhing response induced by acetic acid is an acute pain model that is easy to learn, replicable, and fast to perform, which does not require any special equipment^51^. An acute visceral pain response is induced by i.p. injections of acetic acid into the abdomen^52^. The writhing response is significantly inhibited by intrathecal pretreatment with minocycline at between 10 and 20 min after acetic acid administration, which indicates that spinal microglia are activated in this visceral pain model^15^. In this study, we found that paeonol 10 mg/kg had no effect on acetic acid-induced abdominal contractions, whereas paeonol 30 mg/kg or minocycline 10 mg/kg slightly inhibited contractions (see Fig. 7). Notably, pretreatment with minocycline 10 mg/kg plus paeonol 30 mg/kg significantly decreased writhing numbers to 38.2% of controls. Similarly, pretreatment with paeonol 100 mg/kg or minocycline 40 mg/kg also decreased writhing numbers to 57.7% and 28.8%, respectively, of controls. These results suggest that minocycline and paeonol significantly reduced acute acetic acid-induced visceral pain, which may be related to inhibition of microglial activation.

At 6 h after i.p. injection of LPS, rats or mice are hyperalgesic to heat, mechanical or acetic acid stimuli^35,36^. In the present study, we also found that spinal astrocyte and microglia underwent morphological changes at 6 h after i.p. injection of LPS in spinal dorsal horn (Supplementary Fig. 1 and Fig. 2), and Western blot analysis shows that LPS injection significantly increased relative expression levels of Iba1 and GFAP at 6 h compared to those in controls, in mouse spinal cord (Supplementary Fig. 3). Similarly, Western blot analysis also reveals an increased expression of Iba1 in rat spinal cord at 6 h after LPS treatment, while minocycline pretreatment (i.p.) significantly reduces LPS-induced elevations in Iba1 levels and suppresses LPS-induced hyperalgesia^35^. These results indicate that LPS-induced hyperalgesia may be mediated by activation of microglial cells in the spinal cord. In this study, pretreatment with LPS (100 µg/kg) 6 h before acetic acid injection increased the number of acetic acid-induced writhes by 142.6% compared to controls (see Fig. 8). In mice pretreated with minocycline (40 mg/kg) at 1 h or paeonol (100 mg/kg) at 20 min before LPS (100 µg/kg) administration, the number of writhes induced by acetic acid 0.6% was significantly decreased to 61.5% and 54.8%, respectively, compared to the LPS plus acetic acid group. The reduction in LPS-induced hyperalgesia may relate to the inhibition of microglial activation by pretreatment with minocycline or paeonol. Notably, pretreatment with paeonol (100 mg/kg) or minocycline (40 mg/kg) 6 h prior to acetic acid injection did not reduce acetic acid-induced visceral pain (see Fig. 8), due to their loss of analgesic efficacy after this period of time.

## Conclusions

It is known that inhibiting microglial activation can reduce pain, but the precise mechanisms by which microglial activation contributes to nociceptive synaptic transmission is not yet fully understood. In this study, we found that LPS-activated microglia rapidly release ATP, which stimulates astrocyte P2Y1Rs to release glutamate, triggering presynaptic mGluR5 receptors and increasing presynaptic glutamate release, leading to an increase in mEPSC frequency and enhancement of nociceptive transmission. We consider these effects can serve as a new electrophysiological model for evaluating pain and we hypothesize that if drugs can inhibit the LPS-induced enhancement of mEPSC frequency in SDH neurons, they will have analgesic effects by inhibiting microglial activation and reducing nociceptive transmission. We selected the microglial inhibitors minocycline and paeonol as test drugs for this electrophysiological pain model. These agents effectively inhibited LPS-induced enhancement of mEPSC frequency in SDH neurons. In our behavior study, we found that minocycline and paeonol reduced acute acetic acid-induced visceral pain and LPS-induced hyperalgesia, which may be associated with an inhibition in spinal microglial activation. This electrophysiological pain model provides a fast and economic approach for the testing of new analgesic agents through inhibiting microglial activation and enhancing nociceptive transmission.

## Materials and Methods

Prior to the study, approval was granted by the China Medical University Institutional Animal Care and Use Committee, as according to guidelines on the care and use of laboratory animals issued by the Chinese Taipei Society of Laboratory Animal Sciences.

### Electrophysiology

Spinal cord slices were obtained from 6- to 14-day-old Sprague Dawley male rats. After rats were anaesthetized and decapitated, lumbar spinal cord segments were removed. The blocks were glued to the cutting chamber of a tissue slicer (DTK-1000, Dosaka, Kyoto, Japan) with cyanoacrylate glue. Transverse spinal slices through the L4 and L5 segments (200 µm) were dissected and were left to equilibrate in an artificial cerebral spinal fluid (aCSF) at room temperature for at least 1.5 h before undergoing recording. The aCSF consisted of (mM): NaCl 119, KCl 2.5, CaCl_2_ 2.5, MgCl_2_ 1.3, NaH_2_PO_4_ 1, NaHCO_3_ 26.2 and glucose 11, and was oxygenated with 95% O_2_/5% CO_2_ (pH = 7.4)^34,53^.

We placed individual slices perfused with ACSF at 1.5 mL/min at room temperature in a chamber mounted in an upright microscope. The patch pipettes were filled with solutions containing (in mM): 131 potassium gluconate, 20 KCl, 8 NaCl, 10 HEPES, 2 EGTA, 2 ATP, and 0.3 GTP (pH 7.2 to 7.3) with resistance levels of 6~10 MΩ^34,53^. In laminae I–II of the spinal SDH neurons, whole-cell electrophysiological signals were recorded using a Multiclamp 700B amplifier (Molecular Devices). The Multiclamp 700B amplifier measured mEPSc signals, which were low-pass filtered at 2 kHz, sampled at 5–10 kHz and processed by a Digidata 1440A AD converter, then analyzed by the Clampfit 10 software system. We used MiniAnalysis software (Synaptosoft, NJ, USA) to manually select the mEPSCs and count the amplitudes and frequencies. Considering the noise level from our recording conditions, we set the parameter to “detection threshold” at 10 pA and adjusted the parameter “period to average a baseline” from 1000–6000 µs to reduce noise levels.

Resting membrane potential (RMP) and series resistance (Rs) values were monitored throughout the experiment. The Rs values ranged from 8 to 20 MΩ. Neurons expressing changes in the Rs of over 35%, or that had an RMP negativity exceeding –45 mV at the end of each experiment, were excluded from further analysis^54^.

### Behavioral assessment

Behavioral experiments with mice were conducted in a quiet testing room by an investigator blinded to drug treatment.

### Acute visceral pain induced by acetic acid

Acetic acid produces distinctive abdominal constrictions in ICR mice, consisting of muscle contraction as well as hind limb stretching^52,55^. In this experiment, ICR mice were either given i.p. injections of minocycline 10 mg/kg or 40 mg/kg 1 h before an i.p. injection of acetic acid (0.6% v/v, diluted in saline, 10 mL/kg)^37,56^, or they received i.p. paeonol 10 mg/kg, 30 mg/kg or 100 mg/kg, 20 minutes before the acetic acid injection^57^. The number of abdominal contractions (writhing moments) was counted for 15 min, starting 5 min after acetic acid injection.

### LPS-induced hyperalgesia

It is well established that 6 h after LPS administration, rodents are hyperalgesic to heat, mechanical or acetic acid stimuli^35,36^. Previous research has established that mice injected intraperitoneally with LPS at a dosage of 100 µg/kg sustain hyperalgesia without associated adverse effects, such as cachexia, diarrhea, or sustained tumbling^36^. We therefore used the LPS dosage of 100 µg/kg (i.p.) to induce hyperalgesia. Hyperalgesic responses to LPS in the presence or absence of minocycline or paeonol were assessed by acetic acid-induced abdominal constriction (writhing) testing.

### Compounds

LPS, minocycline, paeonol, MRS2179 ammonium salt hydrate, MPEP hydrochloride, bicuculline methiodide, DL-2-amino-5-phosphonovaleric acid (APV), and 6-cyano-7-nitroquinoxaline-2,3-dione (CNQX) were purchased from the Sigma Chemical Company (St Louis, MO, USA), sodium fluoroacetate was purchased from Chem Service, Inc. (West Chester, USA), (N)-methanocarba-2MeSADP (MRS2365) was purchased from R&D Systems, Inc. (Minneapolis, USA), and tetrodotoxin (TTX) was purchased from Alomone Labs Ltd. (Jerusalem, Israel). Paeonol was prepared in ethanol; MPEP and CNQX were prepared in DMSO; distilled water was used for all other drug stocks.

### Data analysis

Statistical significance of between-treatment differences was determined by the Student’s *t*-test; the paired *t*-test was used to determine the statistical significance of any within-treatment differences. Statistical analysis was carried out using the Statistical Package for Social Sciences (SPSS) version 18 package for Windows (Chicago, IL). Data are expressed as the mean ± S.E.M; “n” indicates the number of neurons tested. Differences were considered significant at p < 0.05.

## Supplementary information

Supplementary Information
