# Supplementary material for: Inhibiting the LPS-induced enhancement of mEPSC frequency in superficial dorsal horn neurons may serve as an electrophysiological model for alleviating pain
Source: Sci Rep. 2019 Nov 5;9:16032. doi: 10.1038/s41598-019-52405-0 (PMC6831605; doi:10.1038/s41598-019-52405-0)
Supplement: Supplementary file 1 — Supplementary Information [file 41598_2019_52405_MOESM1_ESM.pdf]

Supplementary Information

Inhibiting the LPS-induced enhancement of mEPSC frequency in superficial dorsal horn neurons may serve as an electrophysiological model for alleviating pain

Chin-Tsang Yang[1,2], Shih-Ya Hung[3,4,6], Sheng-Feng Hsu[5,6], Iona MacDonald[6], Jaung-Geng Lin[1], Sih-Ting Lu[6], Pei-Lin Lin[7*], Yi-Hung Chen[6,8,9*]

[1]School of Chinese Medicine, China Medical University, Taichung, Taiwan. [2]Department of Leisure Industry and Health Promotion, National Ilan University, Yilan, Taiwan. [3]Department of Medical Research, China Medical University Hospital, Taichung, Taiwan. [4]Graduate Institute of Integrated Medicine, College of Chinese Medicine, China Medical University, Taichung, Taiwan. [5]Department of Chinese Medicine, China Medical University Hospital, Taipei branch, Taipei, Taiwan. [6]Graduate Institute of Acupuncture Science, China Medical University, Taichung, Taiwan. [7]Department of Anesthesiology, National Taiwan University Hospital, Taipei, Taiwan. [8]Chinese Medicine Research Center, China Medical University, Taichung, Taiwan. [9]Department of Photonics and Communication Engineering, Asia University, Taichung, Taiwan

*Corresponding author:
Professor Yi-Hung Chen, Graduate Institute of Acupuncture Science, China Medical University, No.91, Hsueh-Shih Road, Taichung 40447, Taiwan. E-mail: yihungchen@mail.cmu.edu.tw
Or
Dr. Pei-Lin Lin, Department of Anesthesiology, National Taiwan University Hospital, Taipei City, Taiwan. E-mail: pll5611@ntu.edu.tw

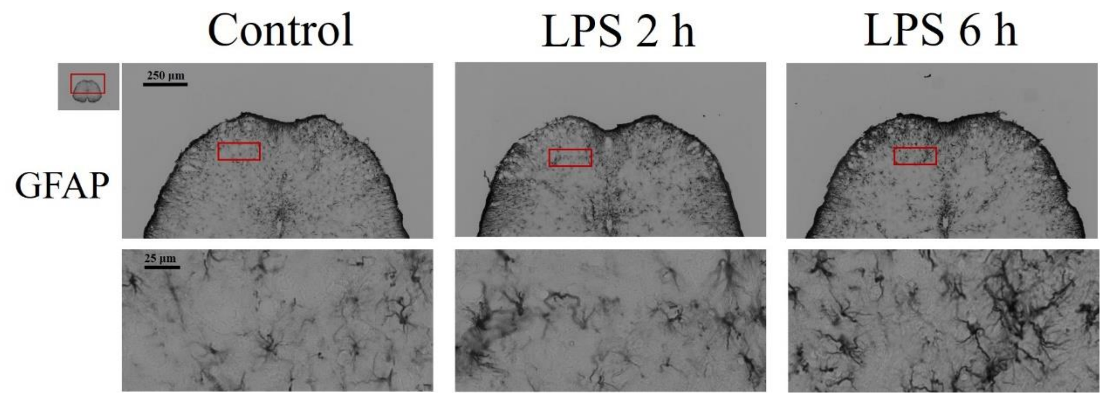

Supplementary Figure 1. Morphological changes of astrocytes induced by LPS in mouse lumbar spinal cord slices (L1–L2). Photomicrographs were taken of control slices (saline injection only) and of slices at 2 h and 6 h after addition of intraperitoneal (i.p.) LPS (100 µg/kg). Glial fibrillary acidic protein (GFAP)-positive cells were imaged at lower magnification (100x) in the upper row and higher magnification (800x) in the lower row. Scale bars show 250 µm in the upper row and 25 µm in the lower row.

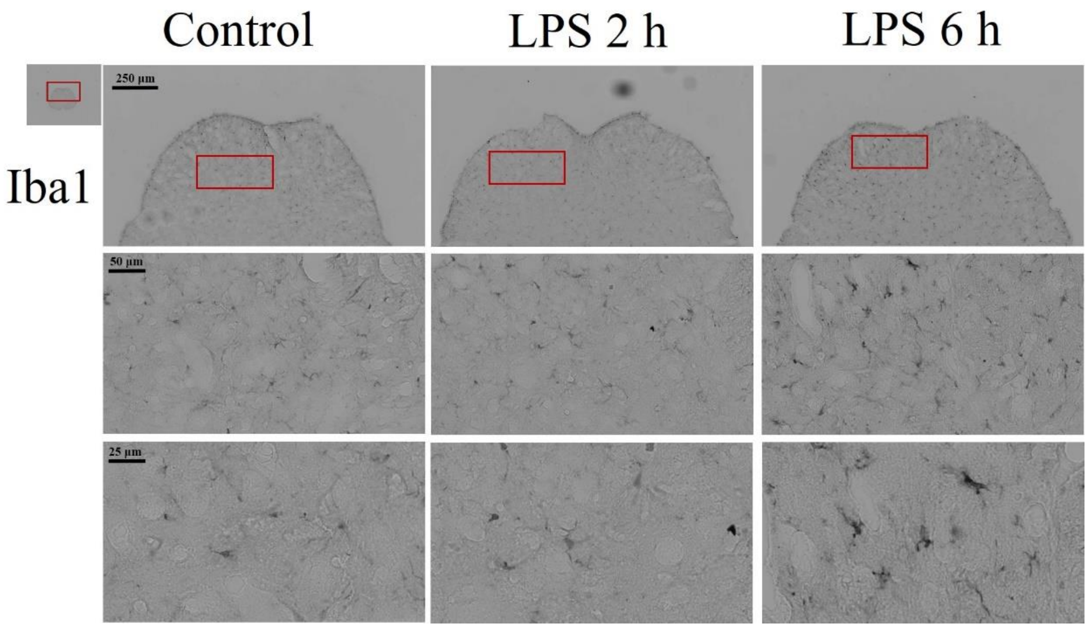

Supplementary Figure 2. LPS-induced morphological changes in microglia from mouse lumbar spinal cord slices (L1–L2). Photomicrographs were taken of control slices (saline injection) and slices at 2 and 6 h after addition of LPS (100 µg/kg; i.p.). Ionized calcium-binding adaptor molecule 1 (Iba1)-positive cells were imaged at 100, 400, and 800 magnifications in the upper, middle and lower rows, respectively. Scale bars show 250 µm in the upper row, 50 µm in the middle row and 25 µm in the lower row.

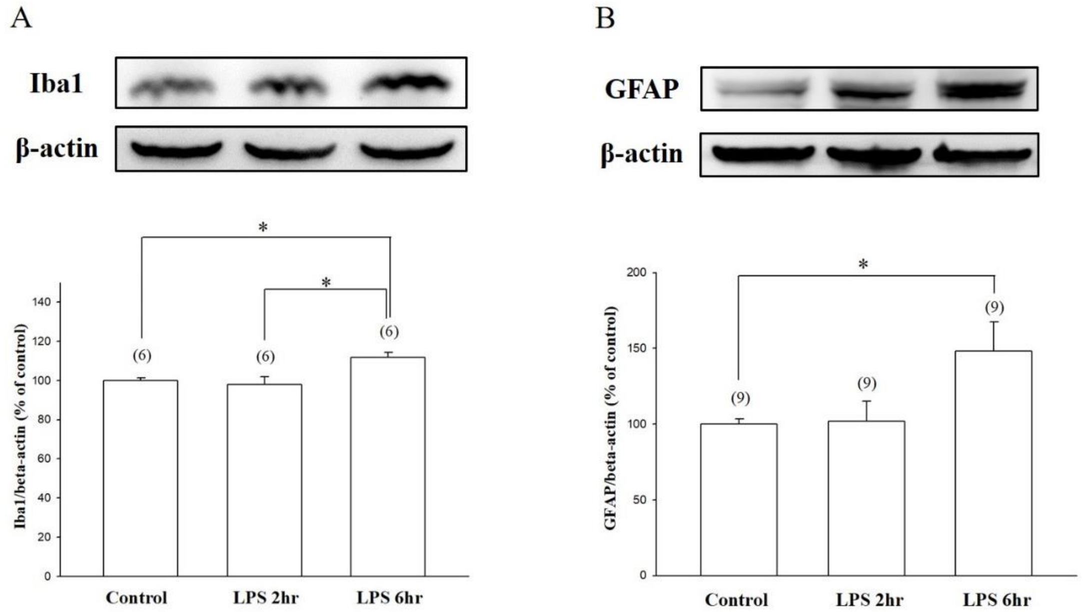

Supplementary Figure 3. Western blot and quantitative analysis illustrating the effects of LPS (100 μg/kg) injection at different time points (2 h and 6 h) on relative expression levels of (A) Iba1 and (B) GFAP, in spinal cord tissue.

Supplementary Methods

We injected saline or LPS (100 μg/kg; i.p.) into mice and collected the spinal cords at different time points (2 h and 6 h after LPS application), then performed Western blot and immunohistochemistry (IHC) staining with GFAP and Iba1 antibody (astrocyte and microglia markers, respectively).

Western blot

Iba1 and GFAP protein levels were determined by Western blot analysis using previously reported methods [1,2], with some modifications. Spinal cord segments were extracted from cervical L1–L2. Tissue samples were homogenized in lysis buffer containing protease inhibitors and phosphatase inhibitors. Each sample of protein concentration was evaluated using a Pierce BCA Protein Assay Kit. Proteins (25 μg) were resolved by 8–12% sodium dodecyl sulfate-polyacrylamide gel electrophoresis (SDS-PAGE) under reducing conditions and the gel was transferred to polyvinylidene difluoride (PVDF) membranes, then incubated overnight at 4°C with Iba1 (1:2000; ab5076; Abcam) antibody or GFAP (1:2000; 3670; Cell Signaling Technology) diluted in Tris-buffered saline (TBS) and probed with β-actin antibody (1:10000; GTX629630; GeneTex) as a standard. Membranes were incubated with secondary antibody (1:10,000; anti-mouse IgG-HRP sc-2005; or anti-goat IgG-HRP sc-2020;

Santa Cruz). Protein bands were detected and estimated.

Immunohistochemistry evaluations

Immunohistochemistry evaluation was performed according to our previously described methods with some modification [2,3]. Mice were anesthetized with i.p. injections of urethane (1.2 g/kg; Sigma-Aldrich) and perfused intracardially with chilled 0.1 M phosphate-buffered saline (PBS) followed by 10% formalin. The lumbar spinal cord was removed and fixed in a 10% formalin solution for 2 days at 4°C. Tissue samples were transferred to 30% sucrose/PBS solution for at least 1 day before sectioning. Transverse spinal sections of 30 μm were prepared at –25°C using the frozen section procedure. Spinal sections were treated with 3% hydrogen peroxide to eliminate endogenous peroxidase activity prior to incubation with an antibody against Iba1 (polyclonal rabbit anti-Iba1; 1:400 dilutions; Wako Chemicals USA, Richmond, VA, USA) and antibody against GFAP (polyclonal mouse anti-GFAP; 1:300 dilutions; Wako Chemicals USA, Richmond, VA, USA). Sections were incubated with 10% antiserum and 0.1% TritonX-100 in PBS, followed by 1 h with biotinylated secondary antibody, then for 30 min with avidin-biotin-peroxidase complex (ABC) kits (Vector Laboratories). Finally, sections were labelled with 0.01% hydrogen peroxide and 0.05% 3,3`-diaminobenzidine (Sigma-Aldrich), then mounted on slides with 0.25% gel alcohol, air-dried and dehydrated with graded ethanol (50%, 70%, and 95% for 6 min each) followed by 100% ethanol for 10 min and xylene (3 times, for 10 min each time). The stained sections were scanned with a NanoZoomer-XR digital slide scanner (Hamamatsu, Hamamatsu City, Japan) and processed by its viewing platform (NDP.view2).

References

1 Lee, Y.-C. *et al.* Manual acupuncture relieves bile acid-induced itch in mice: the role of microglia and TNF-α. *Int. J. Res. Med. Sci.* **15**, 953 (2018).

2 Lin, J. G. *et al.* Electroacupuncture inhibits pruritogen-induced spinal microglial activation in mice. *Brain Res.* **1649**, 23-29 (2016).

3 Hung, S. Y. *et al.* Overexpression of heme oxygenase-1 protects dopaminergic neurons against 1-methyl-4-phenylpyridinium-induced neurotoxicity. *Mol. Pharmacol.* **74**, 1564-1575 (2008).
